# Objectively and Subjectively Measured Physical Activity and Their Associations With Cardiometabolic Risk in the UK Biobank: Retrospective Cohort Study

**DOI:** 10.2196/54820

**Published:** 2025-08-27

**Authors:** Charlyne Bürki, Caiwei Tian, Kenneth Westerman, Chirag Patel

**Affiliations:** 1 Department of Biomedical Informatics, Harvard Medical School, 10 Shattuck St, Boston, MA, 02115, United States, 1 6174321195; 2 Department of Biosystems Science and Engineering, Swiss Federal Institute of Technology Lausanne, Lausanne, Switzerland; 3 Faculty of Arts and Sciences, Harvard University, Cambridge, MA, United States; 4 Clinical and Translational Epidemiology Unit, Mongan Institute, Massachusetts General Hospital, Boston, MA, United States; 5 Programs in Metabolism and Medical Population Genetics, Broad Institute of MIT and Harvard, Cambridge, MA, United States

**Keywords:** accelerometer, exercise, risk factor, sedentary, biobank, physical activity, cardiometabolic risk, cardiometabolic, observational study, clinical biomarker, biomarkers, body mass index, pulse rate, glucose, cholesterol

## Abstract

**Background:**

The association between physical activity (PA) behavior and cardiometabolic risk factors has depended largely on questionnaire-based reporting. More studies are turning to mobile health (mHealth) device solutions to measure PA. While there are differences between self-reported activity levels and objectively measured accelerometer-based activity, how these differences manifest in disease risk is unknown.

**Objective:**

Here, we sought to evaluate these differences between self-reported and mHealth-based PA and to model the impact on their association with cardiometabolic factors. Our study provides a framework to assess the quality of relationships measured by mHealth technologies, which is generalizable to other sensors or activity-measuring devices.

**Methods:**

We assessed PA using both wrist-worn accelerometer data and self-reported questionnaires in 16,000 participants of the UK Biobank (UKB) between 2013 and 2015, focusing on walking, sleeping, sedentary, and moderate-to-vigorous physical activity (MVPA). We compared the concordance between self-reported and objective measures of PA. We also compared the association between objectively measured or self-reported PA and future clinical biomarker levels (eg, BMI, pulse rate, glucose control, and cholesterol).

**Results:**

Participants underestimated their weekly sedentary duration on average of 2.86 hours, and the coefficient of correlation (*r*) between subjective and objective activity was 0.12 for sedentary time, 0.16 for MVPA, 0.18 for walking, and 0.13 for sleeping. We found an inverse association between objectively measured MVPA and cardiometabolic biomarkers such as BMI and pulse rate, but found no association between subjectively reported activity and cardiometabolic biomarkers. We estimated that there is a 6% larger association between subjectively measured MVPA and BMI in healthy adults (vs the objective counterpart). We also estimated a 2%-3% difference on a healthy adult heartbeat (healthy range: 60-100 bpm) if relying on subjectively reported observations instead of measured PA.

**Conclusions:**

These findings suggest that the association based on self-reported activity is likely overestimated and biased compared with objectively measured PA. Therefore, care should be taken when assessing the effects of self-reported PA on key cardiometabolic factors, such as BMI and pulse rate. We emphasize that while the associations are biased when comparing PA modalities, we cannot conclude which method more closely reflects the daily activity load.

## Introduction

It is well established that physical activity (PA) is paramount in the maintenance of health and reduction of risk of chronic disease [1]. PA, whether self-reported or measured by mobile health (mHealth) devices, is consistently associated with higher risks of cardiovascular diseases, type 2 diabetes, low life satisfaction, and general worse health [2-5]. A better understanding of the relationship between specific PA behaviors and health is critical for disease prevention, but efforts are hindered by the complexity of PA assessment.

PA is characterized by 4 dimensions (type, frequency, duration, and intensity), is carried out in at least 4 different contexts (home activities, leisure, transport, and occupational), and can be reported in at least 3 different manners (using energy expenditure in kilocalories, categorizing time spent in different types of activities over a period of time, or using the metabolic equivalent [MET] of a task) [6]. The heterogeneity in reported PA contributes to the conflicting results in this field and adds to the difficulty of discerning a clear link between PA and diseases or biomarkers predictors of diseases. For instance, in the US Centers for Disease Control and Prevention National Health and Nutrition Examination Survey (NHANES), Troiano et al [7] found that less than 5% of US adults met PA directives when relying on accelerometer-measured PA, but more than 51% did when basing on self-reported questionnaire data. Using the same survey, Atienza et al [8] found that physiological and anthropometric biomarkers associated more strongly with accelerometer-derived moderate-to-vigorous physical activity (MVPA) measurements than self-reported PA. Other meta-analyses also reported systematic underestimations of measured sedentary time (as much as 1.74 hours per day) when using self-reported measures [9,10]. As a result, there is conflicting evidence for the validity of relying on self-reported PA.

Sensors and wearable devices (mHealth devices), including accelerometric devices, have progressively integrated into cohort studies, allowing an increasingly diversified field of participants and an easier framework to collect measured data. While these wearable devices can measure PA, they cannot determine disease risk outcomes directly from measured PA. Instead, physiologically relevant metrics modulating diseases must be measured.

Then, a relationship between measured (mHealth) and self-reported PA levels and disease can be quantitatively compared. If these are assessed in the same cohort study, then it is possible to determine whether mHealth devices allow obtaining additional signals with lower error in comparison to traditional self-reporting PA. Furthermore, it is also possible to demonstrate the added value of mHealth-like devices for research through such quantitative comparisons.

In this study, we evaluated the concordance between objectively-measured (via mHealth devices) and subjectively-reported PA in their association with biomarkers (eg, BMI, pulse rate) of cardiometabolic disease in the UK Biobank (UKB) cohort for a quantitative comparison of these measurement techniques in a research setting. We examined the differences between objective and subjective activity population means at an individualized level. Finally, we compared the different types of activities in the cohort, highlighting the discrepancies found when predicting cardiometabolic biomarker levels from activity levels. We anticipate that the approaches developed here to systematically study associations between PA data modalities and clinical outcomes will be generalizable to new approaches and sensors that measure activity. Thematically, these findings demonstrate addressing key themes in mHealth, including mHealth for Data Collection and Research, Evaluation and Research Methodology for mHealth, and Fitness Trackers.

## Methods

### Physical Activity Measurements

#### Measured Activity Derived From an Accelerometer

It is hypothesized that weekend and weekday patterns of MVPA are different [5]. Therefore, we sought to analyze the population on both weekends and weekdays separately. Hourly proportion of time spent in weekly and weekend activity was extracted and preprocessed by Le Goallec et al [11] from the publicly available software [12,13] and converted to weekly minutes of types of activity. The weekly total activity of the 4 types of activity—walking, sleeping, sedentary, and MVPA—was obtained by summing all the hourly proportion of weekday and weekend separately, weighting by 5 and 2, respectively, and converted to weekly minutes by multiplying by 60. Following the filtering out of missing or unusable accelerometer-derived data, the number of participants was 16,467. For sensitivity analysis, we also subdivided the study population to only retain individuals who had a nonwear time of zero days, meaning that all the included participants wore the accelerometer device for an uninterrupted period of 7 days. This allowed for a quality assessment of the objective PA in 5587 individuals.

#### Self-Reported Activity Derived From the Physical Activity Questionnaire

Weekly minutes of activity estimated by questionnaires were obtained by multiplying the weekly frequency by the daily amount (see Table S1 in Multimedia Appendix 1 for all the questions) only if participants had answered both questions. The questionnaire answers were part of a short-form International Physical Activity Questionnaire (IPAQ) filled out at every visit. If a participant did not recall the duration of an activity but had at least practiced it for 10 minutes, the participant’s average was substituted. Any participant with an incomplete answer was dropped. To compare to the measured MVPA, weekly minutes of moderate activity were added to vigorous physical activity (VPA) reported by participants. Weekly minutes of sleep were calculated by converting the reported daily hours of sleep to daily minutes and multiplying by 7. Self-reported time spent watching television, on the computer, and driving was added and capped at 7560 weekly minutes (corresponding to 18 daily hours) as other studies have reported doing [3] to obtain weekly sedentary minutes. If participants reported spending less than 1 hour in either category, the total for that category was encoded as 0 minutes.

### Anthropometric and Biomarker Data

#### Response Variable Encoding

We chose “response” variables, or dependent clinical variables that include anthropometrics and plasma biomarkers, such as BMI and pulse rate [3,14,15]. We also chose “negative controls,” such as sunscreen use. Blood pressure readings were averaged for the visit. Answers containing “prefer not to answer” were excluded, and those that answered “do not know” were replaced with the average of the population to maximize the study population size.

#### Covariate Selection to Adjust for Lifestyle and Comorbidities

Dietary factors were included as covariates in regression models incorporated as 6 separate regression terms in the final model, each accounting for weekly intakes of vegetables, fruits, poultry, fish, meat, and bread-type. We included a variable for red meat (eg, pork, beef, lamb/mutton) and another for poultry. Bread type was encoded as a binary indicator of nonwhite or white bread.

We used a modified version of the Charlson Comorbidity Index (CCI), which measures the total comorbidities for a participant, to account for frailty and disease. We also included in the modified CCI any self-reported diabetes diagnosed by a practitioner or a medical record for type I or type II diabetes, any self-reported cancer diagnosed, any *ICD-10* (*International Statistical Classification of Diseases, Tenth Revision*) record for Chronic Obstructive Pulmonary Disease, and any record of major cardiovascular diseases.

### Statistical Analysis

#### Study Population Statistics

We reported the mean and SD for all measures where possible. For proportions, we provided a percentage and the nonsimplified fraction.

#### Quantifying the Measured/Self-Reported Relationship

The median and IQR of each activity, for both self-reported and measured, were calculated. Pearson correlation *r* was reported between measured/self-reported pairs of each activity type.

#### Estimating Associations Between Biomarker Levels and PA in 2 Separate Models for Measured and Self-Reported Activity

Models predicting individual biomarker levels from self-reported PA only (MVPA and sedentary time) were fit and adjusted for self-reported dietary intake, modified CCI, and smoking status. Separately, we modeled individual biomarker levels from measured PA, for both MVPA and sedentary time, adjusting for dietary intake, adapted CCI, and smoking status. The models using only measured PA values weighted each observation by a decay function that mapped individuals’ time interval between their measurement and the subsequent date at which the biomarker was recorded. This mitigated crosstalk from other factors prone to vary as time passes and affect biomarker levels, such as medication use, by upweighting individuals with biomarker measurements closer to the date of activity measurement. The decay function, $f\left(∆days\right)=\frac{1}{\sqrt{2\pi }}e^{\frac{-∆{days}^{2}}{2{\left(2*365\right)}^{2}}}+0.5$, is a Gaussian with a SD of 2 years, translated vertically by 0.5 units and normalized.

We extracted the β coefficients and their 95% CI of respectively measured and self-reported PA to compare effect sizes for every permutation of biomarker/activity combination, stratifying the results by sex and age. To calculate the average effect size difference between subjectively and objectively reported categories of PA, we simply multiplied the effect sizes by, respectively, the subjective and objective population means of MVPA and sedentary time.

#### Quantifying the Independent Contribution of Objective PA Versus Self-Reported PA

To compare the effect size and the independent contribution of objective versus self-reported PA, we estimated the 95% CI of measured and self-reported estimates. Specifically, we fit a model predicting the biomarker level (“response” variable) as a function of measured PA or self-reported PA, and other adjusting variables. In summary, if the CI of the PA estimate after adjusting for lifestyle and the CCI variables did not contain 0 (was *P* value significant), we concluded that measured PA added additional signal independent of self-reported PA. The impact of PA on selected biomarkers was ranked based on the estimates’ magnitudes.

All statistical analyses were carried out on software R version 4.2.1 (R Core Team) with RStudio 2022.02.1+461 “Prairie Trillium” (Posit, PBC) release for Chrome (Google LLC)/111.0.0.0.

### Ethical Considerations

We applied for internal review board (IRB) approval to the Harvard University IRB. The Harvard University IRB approved of this study (IRB: IRB16-245). The project number for this manuscript is 22881. The UKB investigative team had informed consent, and individuals who have opted out of the study have been removed from the analysis. The consent form is provided on the web [16]. The UKB data are deidentified data. UKB recipients were not directly compensated for participation; however, participants were reimbursed for time and travel. More information can be found here [17].

The UKB is a prospective cohort study of 502,507 recruited between 2007 and 2010 [18]. Participants were asked to participate in follow-up studies (at random). Between 2013 and 2015, a total of 103,661 participants consented to wear a wrist accelerometer for 7 consecutive days [19]. The study participants all took part in subsequent visits between 2013 and 2015, following their participation in the accelerometer measurement.

## Results

### Study Population Description

**Figure 1.**
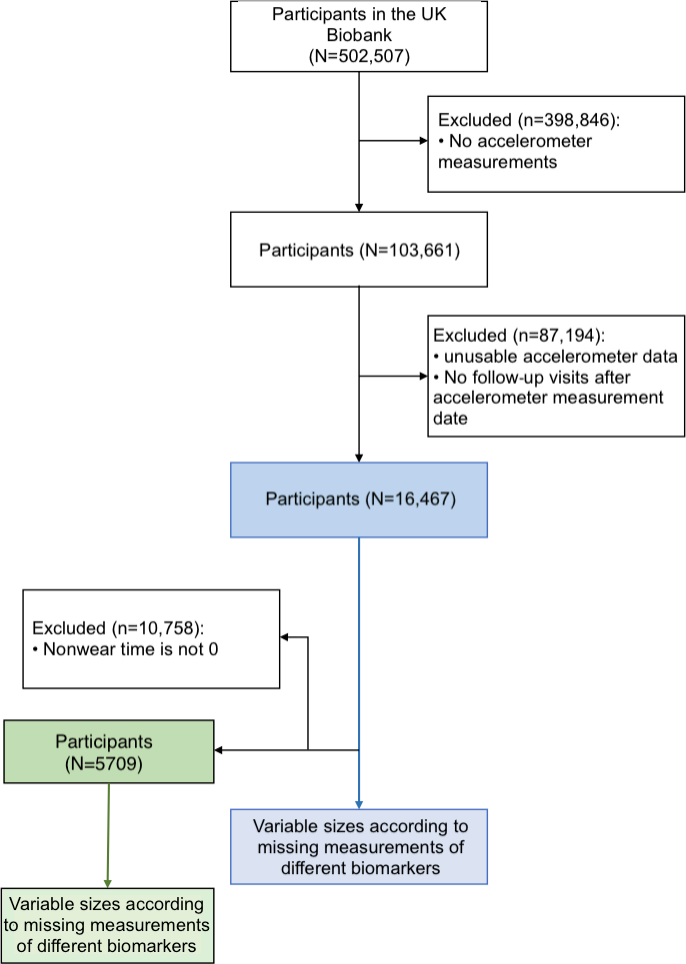
Flowchart of the study: in blue the main study population; in green the subpopulation that wore the accelerometer for an uninterrupted period of 7 days.

Of the 502,507 participants included in the UKB, 103,661 had accelerometer records available. Of those, 16,467 had valid accelerometric data and had anthropometric and biomarker data available following the accelerometer measurement visit (see Figure 1 for the flowchart of the study). The study cohort was comparable to the whole UKB population, in that it was primarily white (97% vs 95%), had similar measured anthropometrics such as BMI (26.29 [4.39] $\frac{kg}{m^{2}}$ vs 27.43 [4.8] $\frac{kg}{m^{2}}$) and similar proportions of males and females (53% females vs 54%). However, the study population was more educated (56% in the highest education category vs 33%), had higher income (31.5% in the top 2 income categories vs 25.4%), and healthier lifestyle habits in that the majority had never smoked (63% vs 55%). The studied cohort was also on average 4 years older than the whole UKB population at baseline, even after accounting for the time elapsed between initial recruitment and the time of measured PA. Refer to Table 1 for all evaluated characteristics.

**Table 1.** Descriptive data comparing the study population with the UK Biobank cohort.

Characteristics	All, (N=502,406)^a^	Study, (N=16,467)^b^
Sex, n (%)		
Female	273,324/502,406 (54)	8726/16,467 (53)
Male	229,082/502,406 (46)	7741/16,467 (47)
Ethnic background		
Bangladeshi	236/499,630 (<0.1)	6/16,425 (<0.1)
Black	8058/499,630 (1.6)	90/16,425 (0.5)
Chinese	1573/499,630 (0.3)	50/16,425 (0.3)
Indian	5951/499,630 (1.2)	97/16,425 (0.6)
Mixed	2954/499,630 (0.6)	71/16,425 (0.4)
Other Asian	1857/499,630 (0.4)	20/16,425 (0.1)
Other ethnic group	4557/499,630 (0.9)	71/16,425 (0.4)
Pakistani	1835/499,630 (0.4)	15/16,425 (<0.1)
White	472,609/499,630 (95)	16,005/16,425 (97)
Prefer not to answer, n	2776	42
Age (years), mean (SD)	56.57 (8.10)	64.32 (7.76)
Above 65 years old	74,746/502,406 (15)	6287/16,467 (38)
BMI	27.43 (4.80)	26.29 (4.39)
Missing	3104	517
Smoking		
Never	273,473/499,458 (55)	10,358/16,467 (63)
Previous	173,023/499,458 (35)	5630/16,467 (34)
Current	52,962/499,458 (11)	479/16,467 (2.9)
Prefer not to answer, n	2948	0
Highest education level (years)		
7	84,140/485,067 (17)	634/14,319 (4.4)
10	81,390/485,067 (17)	1558/14,319 (11)
13	26,247/485,067 (5.4)	712/14,319 (5.0)
15	56,956/485,067 (12)	1361/14,319 (9.5)
19	75,217/485,067 (16)	2075/14,319 (14)
20	161,117/485,067 (33)	7979/14,319 (56)
Prefer not to answer, n	17,339	2148
Resting pulse rate	69.36 (11.26)	68.35 (11.54)
Missing, n	30,109	2870
Body fat, n (%)	31.45 (8.55)	30.78 (8.13)
Missing, n	10,405	847
Waist circumference	90.31 (13.49)	87.74 (12.72)
Missing, n	2160	481
Alcohol consumption		
Never	40,627/502,406 (8.1)	1062/16,467 (6.4)
Occasionally	113,836/502,406 (23)	3624/16,467 (22)
Frequently	346,443/502,406 (69)	11,781/16,467 (72)
Prefer not to answer	1,500/502,406 (0.3)	0/16,467 (0)
Household income (in Great British Pounds)^d^		
<18,000	97,180/425,265 (23)	1690/14,849 (11)
18,000-30,999	108,157/425,265 (25)	4053/14,849 (27)
31,000-51,999	110,751/425,265 (26)	4471/14,849 (30)
52,000-100,000	86,250/425,265 (20)	3515/14,849 (24)
>100,000	22,927/425,265 (5.4)	1120/14,849 (7.5)
Prefer not to answer	77,141	1618
Blood pressure (systolic/diastolic)	137.82(18.68)/82.22(10.16)	138.79(18.77)/78.54(10.05)
Missing, n	30,109	2870
Self-reported weekly PA^c^ (mins)		
Moderate to vigorous	362.56 (550.29)	377.58 (463.31)
Sedentary	1,900.43 (1,065.78)	2051.01 (1000.46)
Walking	358.38 (502.41)	358.86 (416.73)
Sleeping	3,006.04 (456.83)	3005.02 (426.04)
Missing, n	46,972	0
Self-reported nutrition		
Daily vegetables (heaped table spoon)	4.88 (3.37)	5.08 (3.27)
Weekly fruits (serving)	3.05 (2.63)	3.20 (2.54)
Weekly fish (serving)	2.27 (1.60)	2.36 (1.57)
Weekly meat (serving)	3.60 (2.24)	3.24 (2.10)
Weekly poultry (serving)	1.92 (1.26)	1.93 (1.29)
Missing, n	893	0

a Measures taken at recruitment.

b Measures taken after the visit wherein physical activity was measured.

c PA: physical activity.

d Great British Pound to US Dollar at the time of the study: US $1.21=GBP 1.

### Relationship Between Measured and Self-Reported PA

**Figure 2.**
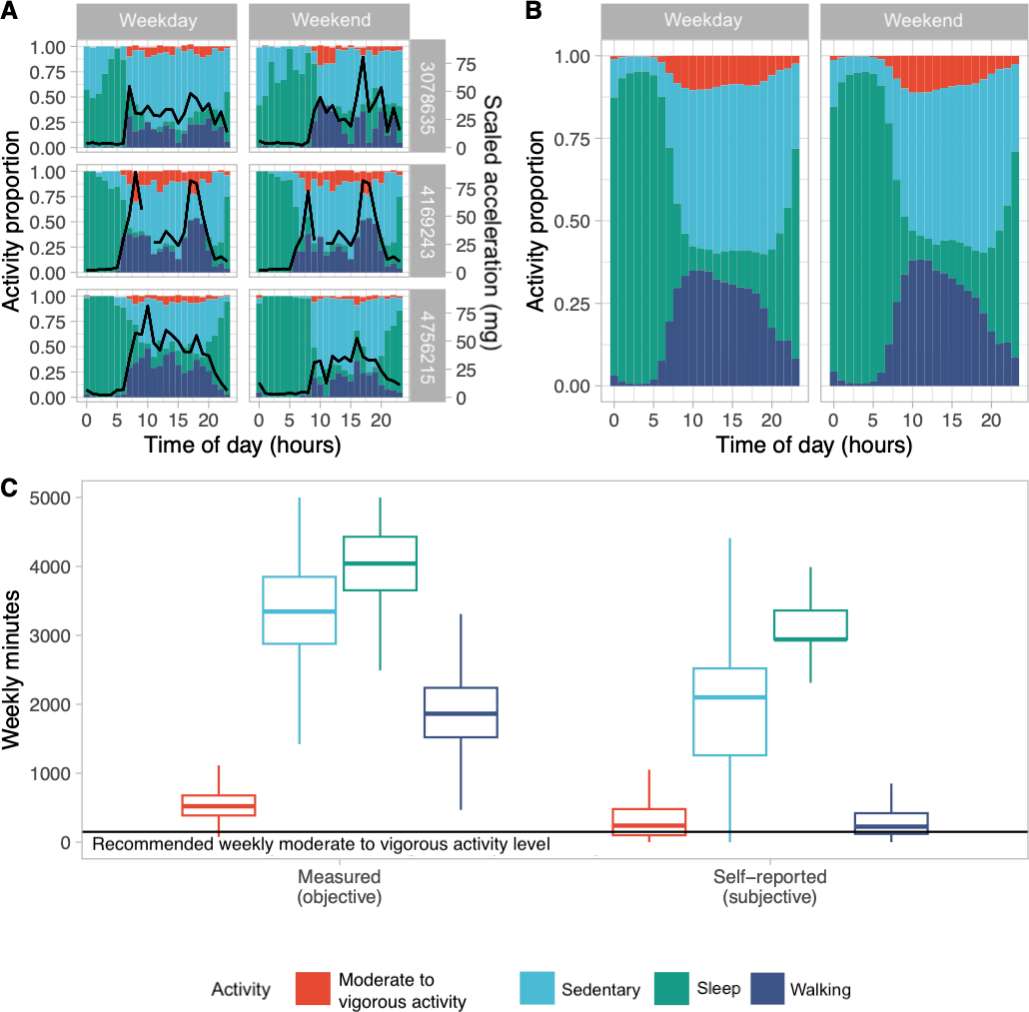
(A) Three individuals’ average measured hourly activity over the course of a day overlaid with their scaled averaged recorded triaxial acceleration (black line) separated by weekday/weekend (black line discontinuities correspond to values in excess of the figure window), (B) the averaged (mean) classified measured hourly activity of the study cohort over a 24-hour period separated by weekday/weekend, and (C) the cohort’s weekly measured and self-reported physical activity, stratified by activity type.

Measured or objective PA was assessed at both the individual level (Figure 2A). We observed concordance between 3 random participants’ hourly classified activities and the averaged triaxial acceleration, and at the population level in Figure 2B. At the population level, and even at the individuals’ level, we found that a little proportion of time was spent in MVPA. Throughout the day, individuals spent at most 12.5% of an hour engaged in MVPA, whereas 50% of their time was spent in sedentary status outside of their sleep. The weekly median number of minutes spent in sedentary time was 3200 minutes (7.62 daily hours), 2000 minutes (4.76 daily hours) in walking, and 500 minutes (1.19 daily hours) in MVPA (Figure 2C). Participants averaged 24-hour period habits during the weekday did not differ from habits during the weekend.

The self-reported levels of sedentary time were not concordant with the objectively measured sedentary time. Participants reported 2000 weekly minutes (4.76 daily hours) of sedentary time, or about 1200 weekly minutes (2.85 daily hours) less than the measured level. The population mean of self-reported walking was similar to the self-reported MVPA population mean, each averaging 250 weekly minutes (0.6 daily hours). However, these self-reported values deviated from their measured counterparts by 1750 minutes for walking (4.17 daily hours) and 250 minutes for MVPA (0.60 daily hours; Figure 2C).

### Quantifying the Measured/Self-Reported PA Relationship

**Figure 3.**
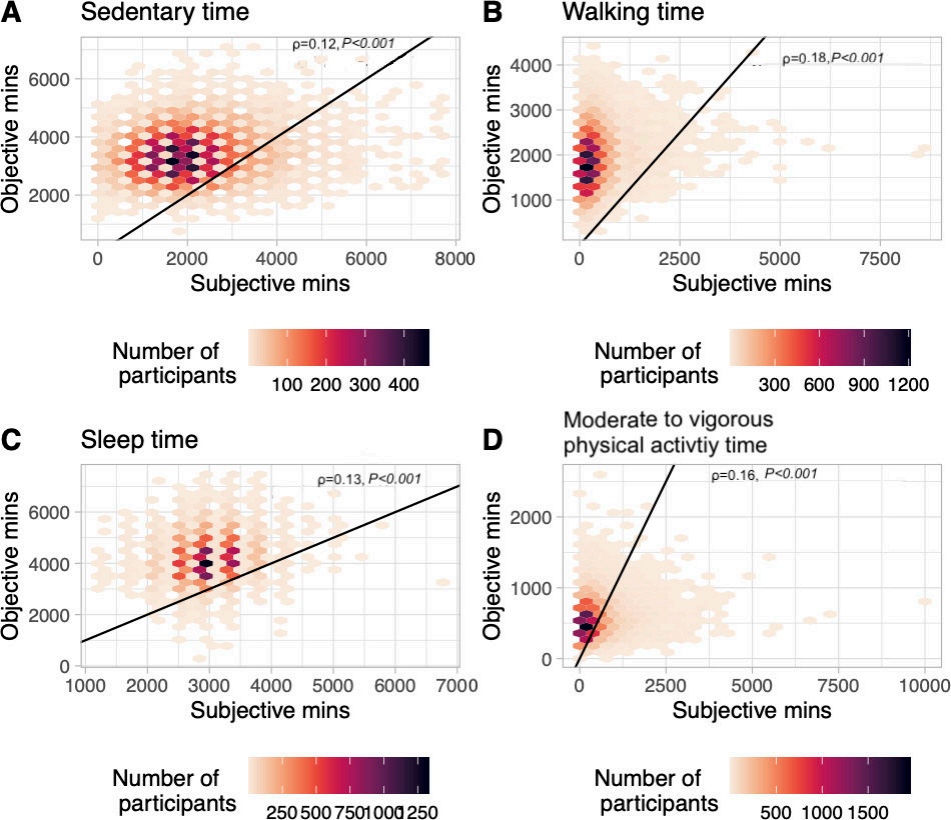
Scatter plots of the study population comparing the reported subjective minutes to the objectively measured minutes in each type of physical activity. The correlation coefficient (*rho*) is reported in each grid. The black line is the identity line (y=x), corresponding to a perfect one-to-one match. Hexagons are color-coded by their density: (A) weekly minutes of sedentary time, (B) weekly minutes of walking, (C) weekly minutes of sleeping, and (D) weekly minutes of moderate to vigorous activity.

Objective PA was modestly associated with subjective PA in Figure 3A-D (*r*=0.12; *P*<.001 for sedentary; *r*=0.18; *P*<.001 for walking; *r*=0.16; *P*<.001 for MVPA; and *r*=0.13; *P*<.001 for sleeping). Interestingly, the correlation was higher for walking and MVPA activities than for sedentary time and sleeping. We found the same correlations for the subpopulation that had noninterrupted accelerometer wear time during the study period (figures not included).

### Effect of PA on Cardiometabolic Biomarkers 


**Figure 4.**
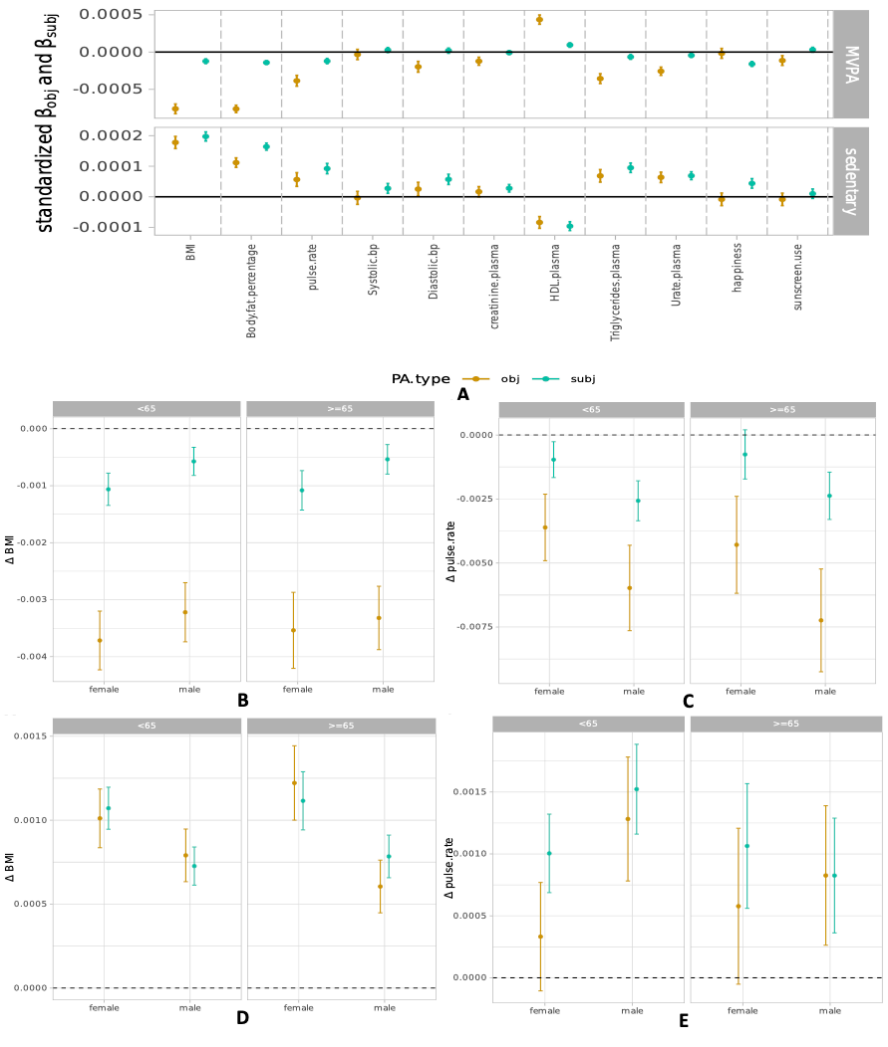
(A) Standardized effect sizes of objective physical activity adjusted for dietary intake, adapted CCI, and subjective physical activity on selected biomarkers. The subjective physical activity coefficients are plotted as a comparison; (B-E) effect sizes of PA on selected biomarkers issued from two separate models of objective and subjective physical activity, stratified by age group: (B) moderate-to-vigorous physical activity BMI, (C) moderate-to-vigorous physical activity pulse rate, (D) sedentary BMI, (E) and sedentary pulse rate.

Figure 4A depicts the standardized effect size of measured and self-reported MVPA and sedentary time on selected cardiometabolic-related biomarker or “response” levels (see Figure S1 in Multimedia Appendix 1). The subjective and objective PA are modeled jointly, such that each measurement’s effect estimate assumes the other is held constant; in other words, the coefficients measure the effect of one PA measure independent of the other. The objective PA association sizes were greatest for anthropometric markers (BMI, waist circumference, body fat mass), certain physiological markers such as pulse rate, and certain plasma biomarkers such as high-density lipoprotein and triglycerides. As a negative control, objective sedentary time did not predict other biomarkers presumably unrelated to physical fitness, such as sunscreen usage (β=−8.42e-6; 95% CI −2.89e to 5,1.20e0; *P=*.42); however, objective MVPA was nominally significant (β=−1.13e−4; 95% CI −1.79e−4 to 4.72e−5; *P*=.009). Also, BMI and pulse rate levels had an inverse association with MVPA (eg, lower BMI and pulse rate for higher levels of MVPA) and a positive association with sedentary time. Further, subjective MVPA estimates were null across all biomarkers, but subjective sedentary time estimates were similar to the estimates predicted by objective sedentary time.

The association sizes of objective MVPA were directionally consistent and stronger in magnitude than those of subjective MVPA in all groups (above/below 65, males/females; Figures 4B and C, Figure S2 in Multimedia Appendix 1). There was also no substantial difference in effect size between the age groups.

In contrast to MVPA, objective and subjective effect sizes for sedentary time coincided almost perfectly for all anthropometric and physiological measurements (see Figure S3 in Multimedia Appendix 1 for all tested biomarkers). Sedentary time was associated with an increase in BMI and pulse rate (Figures 4D and E). We also replicated these tests in the subpopulation that wore the accelerometer without interruption and found the same results (figures not included).

Finally, we calculated the differences of biomarker effect sizes between each subjective/objective PA pair stratified by sex and age (above/below 65 years old) in original and nonstandardized units (eg, beats per minute for pulse rate and $\frac{kg}{m^{2}}$ for BMI). For BMI estimations from weekly MVPA, we found that subjective MVPA was associated with an increase of 1.5 $\frac{kg}{m^{2}}$ more than objective MVPA (1.59$\frac{kg}{m^{2}}$ [1.41, 1.78] for females <65, 1.47$\frac{kg}{m^{2}}$ [1.27, 1.67] for males <65, 1.5 $\frac{kg}{m^{2}}$[1.25, 1.75] for females >=65, and 1.53$\frac{kg}{m^{2}}$ [1.31,1.74] for males >=65). We also found that subjective MVPA was associated with an increase of approximately 2 bpm more than objective MVPA (1.56 bpm [1.09, 2.04] for females <65, 2.34 bpm [1.71, 2.98] for males <65, 1.95 bpm [1.25, 2.66] for females >=65, and 3.03 bpm [2.25, 3.8] for males >=65). For BMI predicted by sedentary time, we found that subjective estimates were lower than objective measurements by, on average, 1$\frac{kg}{m^{2}}$ for all subsets. See Figure S4 in Multimedia Appendix 1 for all tested biomarkers.

## Discussion

### Principal Findings

Here, we quantified the relationship between objective and subjective PA in two ways: (1) in a direct, one-to-one comparison and (2) in their association with cardiometabolic biomarkers. The former yielded similar results to previously reported studies assessing the differences between measured PA assessed with wearable devices and questionnaires [7,10,20,21].

First, the reported low Pearson correlations between objective and subjective PA measures were unsurprising given other reported levels below *r*=0.30 [7,21,22]. A European cohort reported a Pearson correlation of *r*=0.28 [21] while a Norwegian study reported correlations of *r*=0.46 for sedentary time and *r*=0.20 for MVPA [20]. Our results were lower. Further, participants underestimated their sedentary durations by almost 1200 weekly minutes (2.86 hours per day), a larger difference than what Prince et al [10] reported in their systematic meta-study review, where an underestimation of 731 weekly minutes (1.74 hours per day) was reported.

We found great mismatches when matching each individual’s subjective/objective measurements. In particular, we found that the mismatch was greatest in those who self-reported higher values of MVPA. This may have indicated that either participants with high activity levels over-reported their MVPA workout activity, either because (1) the accelerometers were less adapted to validate highly-active participants’ levels, or (2) that other factors like education level, age, or physical ability [20], affected participants’ self-reporting values. A reasonable explanation is probably a combination of these hypotheses. For example, at the population level, measured activity medians of all types were higher than the questionnaire-based ones. In fine*,* discrepancies in perceived PA durations are sure to vary between individuals given their different mobility abilities, which were not assessed in the cohort.

The way that we assess PA has a large influence on the size and robustness of the association with clinical biomarkers of disease. Our findings are in agreement with other studies that describe the inverse association between increased MVPA and BMI and heart rate (respectively, the regular association between sedentary time and BMI and heart rate) [3,14]. However, we observed that objective measurements were more strongly associated with cardiometabolic biomarkers than has been previously appreciated. In the case of increased MVPA, the negative association of measured PA was of a larger magnitude than that of subjective PA by 4e-3 standard units on anthropometric markers and on the order of 1e-3 standard units in the case of plasma biomarkers and pulse rate. In the case of increased sedentary PA, the magnitudes of objective and subjective associations were largely invariant to age and sex/gender.

Moreover, we found discrepancies between levels of BMI and pulse rate estimated with subjective and objective MVPA. Subjective weekly MVPA was estimated on average, in the different population subsets, 1.5 $\frac{kg}{m^{2}}$ and 2 bpm more than objective MVPA. Contextualized in healthy adults, these numbers reflect a 6% overestimation of MVPA association on BMI, if relying on subjectively-reported observations rather than measured MVPA. The MVPA impact on pulse rate differences was less, varying between 2% and 3% difference on an adult heart beating 60-100 bpm. Though small, these variations may present key influences on clinical biomarkers.

### Comparison With Previous Work

Due in part to the ease of implementation of IPAQ (long or short form) or equivalent questionnaires such as 7-day Physical Activity Recall [23], and in part by the relatively new widespread use of wearable devices, most cohort studies still rely on only subjective PA measurements to assess physical fitness. For instance, a recent study using the UKB found that participants adhering to frequent MVPA patterns based on subjective PA were associated with reduced risk of dementia [24]. Given the discrepancies between subjective and objective PA quantified in this study, however, it is questionable whether the reported effect is discernible in objectively measured PA in the UKB cohort.

PA investigation on cardiometabolic disease may benefit from increased power and precision from the use of accelerometer data. These findings support the hypothesis that the difference observed between subjective and objective MVPA measurements was more likely due to misjudging MVPA duration according to fitness levels and not age. Indeed, we did not observe any objective MVPA activity difference between the 2 age groups but found diluted pulse rate associations with increased MVPA activity in both groups (similarly, increased pulse rate with increased sedentary time). Given that the objectively reported effect sizes had greater magnitudes (and perhaps less error) than the subjective ones, greater power may be found with accelerometer-based measurements.

The measured PA data values were extracted and categorized from the freely available software [12,13,19]. Although measured PA was shown to strongly correlate with daily observed and reported behavior, the PA categorizations are only approximations of MET, which is defined as using 3.5 mL of oxygen per minute per kilogram of body weight. By definition, a participant is classified as doing MVPA if the activity duration intensity is greater than or equal to 3 METs. While gold-standard methods such as doubly labeled water [25] exist and result in precise, individual-based calibrations, the method used to process accelerometric data did not benefit from an individual-centric calibration. This, therefore, is one source of the discrepancies between PA classifications.

We hypothesize that individuals with different baseline cardiometabolic health may perceive their PA differently, and this will bias associations between reported activity and cardiometabolic biomarkers. We describe a plausible scenario: for instance, if 2 individuals of different baseline metabolic capacities are carrying out the same activity intensity, such as running a certain distance at a certain pace for a certain time, an individual-centric method would result in different MVPA duration classifications for each individual. Equivalently, an individual with a healthier baseline metabolic capacity would have less MVPA duration than an individual with a lower metabolic capacity. Hence, in a scenario where both individuals run the same distance at the same pace, the accelerometer reading will be identical. However, the self-reported perception of PA may differ; the individual with better cardiometabolic fitness may have perceived and reported doing less MVPA than the other individual. This could translate to the reported discrepancy observed in the study participants and therefore influence the magnitude of the coefficients of the biomarkers studied.

Unlike MVPA, we observed concordant associations between sedentary activity (measured by both accelerometer and self-reported) on biomarker levels. We hypothesize that measurement “error” in the aggregate may be lower for activities related to inactivity, which sum over behaviors such as watching television and using a computer versus reporting one MVPA-related activity carried out weekly [9,20].

With the new generation of mHealth devices that have been developed since the study’s conception, which incorporate personalized thresholds to categorize activity intensities [5]. There is evidence that activity intensity is assessed more objectively and therefore reflects more precise metabolic exertion. Leveraging these threshold personalization allows us to distinguish new behavioral patterns. For instance, McConnell et al [5] identified a cluster of adults who were more active during the weekend (called weekend warriors) in comparison to weekdays. Implementing studies with such devices is, however, expensive, but given the discrepancies highlighted in this study between self-reported and measured PA and predicting cardiometabolic biomarker levels, it may be crucial to re-evaluate PA assessments, especially when these are translated to prevent disease outcomes, such as in guidelines [26]. While we acknowledge the development of new technologies for measuring PA, the core contribution of our paper lies in the method we present to quantify the quality of relationships produced by mHealth technologies. This approach is not limited by specific devices or data sources and, importantly, should generalize to newer technologies as they emerge.

It may be possible, in the future, to create a calibration between self-reported and objective PA to reanalyze results from studies that have one measure and not the other. Many studies rely on self-reported measures. In this study, we are primarily focused on how the discrepancies between self-reported and objective measures impact associations with clinical outcomes, rather than directly translating between the two measures. As we show in Figure 3, the correlation between self-reported and objectively measured PA is very modest, below 0.1, highlighting the challenges inherent in creating such a mapping. This low correlation, in itself, demonstrates that providing a simple or reliable translation will require a formal study and potentially more data.

Use of self-reported versus accelerometer-based approaches for measuring PA has implications in biobank-scale genetic studies, such as “genome-wide association studies” (GWASs) [27]. GWASs are a discovery-based approach to broadly uncover inherited genetic loci, or places along the genome that predispose individuals to perform PA. However, we hypothesize that the scale of genetic discovery (the number of loci found) and their association sizes are influenced by the type of PA being interrogated. A number of papers have begun to interrogate the genetic contributions, via GWAS, in PA using both self-reported [28] and/or objective/accelerometer-based modalities.

For example, Klimentidis et al [29] conducted a GWAS of habitual PA of both self-reported PA and accelerometry data in the same participants analyzed in our study, individuals from the UKB. Briefly, they associated genetic loci with self-reported MVPA and VPA. They also associated genetic loci with average acceleration and fraction of time with higher accelerations as proxies of vigorous activity. In brief, while the sample sizes from each of the GWASs were different, they found different numbers and genetic loci from GWASs of self-reported and accelerometer-based measurement of PA. Specifically, the self-reported and objective indicators had an overlap in some loci found (particularly near gene *Cell Adhesion Molecule 2*), but the concordance was not consistent across all assessments of self-reported and accelerometer-based modalities. At a relaxed genome-wide *P* value threshold, the authors observed 4 genetic loci in common between self-reported MVPA and accelerometer and 3 loci in common between vigorous activity and accelerometer GWASs. Studying the difference in the genetic “architecture,” or the number and biological implications of genetic loci, is an aim for a future study.

### Limitations

Some caution should be used when interpreting these results. First, objective and subjective measurements were not collected at the same time. Participants of the UKB were invited to fill out IPAQ forms during their medical appointments independently of their participation in the accelerometer study. Furthermore, participants in the accelerometer study were not required to schedule a medical appointment during the week of their participation. Thus, self-reported PA was not collected simultaneously with the measured PA. While most participants had self-reported PA measures within weeks of their measured PA recording, some had at most 2 years between the measures. Though we applied a weight correction accounting for time elapsed between the measures (self-reported PA and measured PA) and considered additional lifestyle factors, there were other factors that could not be captured, which may have influenced the observed differences between objective and subjective MVPA.

### Conclusions

In sum, by associating both objective and subjective PA with health indicators and clinical risk variables, we quantified the epidemiological implications of the relationship between subjective and objective PA versus just studying the concordance between objective and subjective PA. We found that BMI and pulse rate followed a positive relation with MVPA and a negative association in the case of increased MVPA. Further, we showed that objective MVPA measurements, even in the presence of subjective MVPA, captured more robust signals than subjective MVPA. Importantly, the framework we presented here allows us to quantify the quality of relationships produced by mHealth versus self-reported approaches. We showed that our comparison should be carried out to evaluate the degree of bias of self-reported versus mHealth devices in large cohort studies. This has implications beyond the accelerometer devices used in this study and is generalizable to other mHealth devices. Thematically, these findings demonstrate addressing key themes in mHealth, including mHealth for Data Collection and Research, Evaluation and Research Methodology for mHealth, and Fitness Trackers.

## Supplementary material

10.2196/54820 Multimedia Appendix 1 Additional table and figures.

## Abbreviations

bpm: beats per minute
CCI: Charlson Comorbidity Index
GWAS: genome-wide association study
*ICD-10*: *International Statistical Classification of Diseases, Tenth Revision*
IPAQ: International Physical Activity Questionnaire
IRB: internal review board
mHealth: mobile health
MVPA: moderate-to-vigorous physical activity
NHANES: Disease Control and Prevention National
PA: physical activity
UKB: UK Biobank
VPA: vigorous physical activity
