# Supplementary material for: Objectively and Subjectively Measured Physical Activity and Their Associations With Cardiometabolic Risk in the UK Biobank: Retrospective Cohort Study
Source: JMIR Mhealth Uhealth. 2025 Aug 27;13:e54820. doi: 10.2196/54820 (PMC12384678; doi:10.2196/54820)
Supplement: Multimedia Appendix 1 [file mhealth-v13-e54820-s001.docx]

**Supplementary Figures**

**Burki et al.**

**Figure S1** standardized effect sizes of objective physical activity on all tested biomarkers in the studied population. The subjective PA coefficients are plotted as a comparison

**Figure S2** a.-h. All tested biomarkers from the model employing moderate to vigorous PA

**Figure S3** a.-h. All tested biomarkers from the model employing sedentary activity

**Figure S2 a.-h.**All tested biomarkers from the model employing moderate to vigorous PA

**Figure S3 a.-h.**All tested biomarkers from the model employing sedentary activity

**Figure S4** The difference of effect sizes from objective and subjective PA on selected biomarkers. The differences are expressed in the units of each individual biomarker (i.e beats per minute for pulse rate). Physical activity is reported as weekly minutes of MVPA or sedentary time, such that the difference in effect size should be read as the difference between subjective and objective PA, after a week of reported PA. The results are stratified by sex and age (above/below 65 years old).

Table S1 Collected questions and field codes

|  | **Question** | **Field code** |
| --- | --- | --- |
| Subjective PA and Sleep | In a typical WEEK, on how many days did you walk for at least 10 minutes at a time? (Include walking that you do at work, traveling to and from work, and for sport or leisure) | 864 |
|  | How many minutes did you usually spend walking on a typical DAY? | 874 |
|  | In a typical WEEK, on how many days did you do 10 minutes or more of moderate physical activities like carrying light loads, cycling at a normal pace? (Do not include walking) | 884 |
|  | How many minutes did you usually spend doing moderate activities on a typical DAY? | 894 |
|  | In a typical WEEK, how many days did you do 10 minutes or more of vigorous physical activity? (These are activities that make you sweat or breathe hard such as fast cycling, aerobics, heavy lifting) | 904 |
|  | How many minutes did you usually spend doing vigorous activities on a typical DAY? | 914 |
|  | In a typical DAY, how many hours do you spend watching TV? (Put 0 if you do not spend any time doing it) | 1070 |
|  | In a typical DAY, how many hours do you spend using the computer? (Do not include using a computer at work; put 0 if you do not spend any time doing it) | 1080 |
|  | In a typical DAY, how many hours do you spend driving? | 1090 |
|  | About how many hours of sleep do you get in every 24 hours? (please include naps) | 1160 |
| Anthropometrics | Body Mass Index (BMI): constructed by height and weight at visit | 21001 |
|  | Body Mass Index (BMI) impedance measurement, encoded as BMI.2 | 23104 |
|  | Waist Circumference | 48 |
|  | Body Fat Percentage | 23099 |
|  | Whole Body Fat Mass (measured by impedance) | 23100 |
|  | Whole Body Fat-Free Mass (measured by impedance) | 23101 |
| Physiological | Systolic Blood Pressure, automated reading, two measurements were taken a few moments apart | 4080 |
|  | Diastolic Blood Pressure, automated reading, two measurements were taken a few moments apart | 4079 |
|  | Pulse rate, automated reading, two measurements were taken a few moments apart | 17518 |
| Blood biochemistry | Albumin | 30600 |
|  | Creatinine | 30700 |
|  | Aspartate aminotransferase | 30650 |
|  | Total bilirubin | 30840 |
|  | Direct bilirubin | 30660 |
|  | HDL cholesterol | 30760 |
|  | LDL direct | 30780 |
|  | Glycated hemoglobin (HbA1c) | 30750 |
|  | Glucose | 30740 |
|  | Triglycerides | 30870 |
|  | Urate | 30880 |
| Dietary intake | Total daily vegetable intake | 1289, 1299 |
|  | Total daily fruit intake | 1309, 1319 |
|  | Total weekly fish intake | 1329, 1339 |
|  | Total weekly meat intake | 1349, 1369, 1379, 1389 |
|  | Total weekly poultry intake | 1359 |
|  | Bread type intake | 1448 |
| Others | Smoking status | 20116 |
|  | General happiness | 20458 |
|  | Alcohol intake frequency | 1558 |
|  | Average total household income before tax | 738 |
|  | Use of sun/UV protection | 2267 |
